# Measurement and Spatial Variation of Green Total Factor Productivity of the Tourism Industry in China

**DOI:** 10.3390/ijerph17041159

**Published:** 2020-02-12

**Authors:** Xingming Li, Pengfei Shi, Yazhi Han, Aimin Deng, Duan Liu

**Affiliations:** 1 Key Laboratory for Geographical Process Analysis & Simulation Hubei Province, Central China Normal University, Wuhan 430079, China; xingming@mail.ccnu.edu.cn (X.L.); hanyazhigeog@163.com (Y.H.); 2 School of Economics and Management, Southwest University, Chongqing 400715, China; 3 School of Business Administration, Zhongnan University of Economics and Law, Wuhan 430073, China; dam99@163.com (A.D.); liuduanwhu@163.com (D.L.)

**Keywords:** tourism industry, green total factor productivity, measurement, spatial variation, China

## Abstract

Promoting tourism in China using sustainable practices has become a very important issue. In order to analyze temporal characteristics and spatial regularities of green total factor productivity (GTFP), carbon emissions and the consumption of energy related to tourism in China were estimated using a "bottom-up" method. The construction of a measurement framework (including carbon emissions and energy consumption) of GTFP for the tourism industry was also undertaken. The data envelopment analysis (DEA) model and the Malmquist–Luenberger (ML) index were used to measure and calculate tourism GTFP in China between 2007 and 2018, as well as analyze spatio-temporal differences. Results indicate that: (1) carbon emissions and the consumption of energy are increasing, and they have not yet peaked, with traffic associated with tourism accounting for the largest proportion among tourism sectors; the spatial distribution of carbon emissions and the consumption of energy is not balanced; (2) green development of tourism in China has achieved a good level of performance during the study period, driven by technical efficiency. Since 2014, pure technical efficiency (PE) has been >1, indicating that the tourism industry in China has entered a stage of change and promotion; (3) significant spatial differences exist in tourism GTFP in China. For example, the overall pattern of being strongest in the east and weakest in the west has not changed. Currently, eastern, central, and western regions in China rely on different dynamic mechanisms to promote tourism green development. In addition, some provinces have become the core or secondary growth poles of tourism green development in China.

## 1. Introduction

Tourism, a national strategic pillar industry, has increased in importance in China as livelihoods have improved and industrial transformation and upgrading have been promoted. However, due to processes and links, tourists not only consume a large amount of energy, they also produce a substantial volume of carbon emissions, therefore resulting in tourism not being a "zero-emission" green industry. In particular, prominent environmental problems associated with the tourist industry in China have arisen due to low efficiency and significant development over a short time period. For example, carbon emissions related to tourism directly and indirectly account for about 5% of total national emissions [1,2], deviating from the original industry characteristics of low source consumption, low environmental pollution, and a sustainable cycle. Therefore, only through the promotion of green development can the tourism industry return to its original industrial characteristics and become a supporting and advantageous industry in the pursuit of sustainable development in China. Tourism green total factor productivity (GTFP) is the direct measure of the green development level of the tourist industry, examining environmental economic output maximization under rigid constraints. GTFP represents the reduction of the environmental impact of tourism and emphasizes the coordination between economic development and the ecological environment [2]. Therefore, measuring tourism GTFP based on the perspective of resources and the environment can enable a more scientific approach to evaluating green development quality and identifying internal laws relating to green development in China’s tourist industry. This will ultimately enable the proposal of certain theoretical guidance for improving the quality of green development.

## 2. Literature Review

GTFP typically represents complete output of input totals under the consideration of resource and environmental constraints. GTFP is also used to analyze the green development quality and sustainable development level of a country or a region [3,4]. Studies using GTFP have made considerable advances recently. For example, the GTFP of energy-intensive and highly polluting enterprises on the micro-level have been examined, with Morfeldt, Long, and Zhang [5,6,7] using a modified data envelopment analysis (DEA) model encompassing the directional distance function to measure the GTFP of steel works, cement plants, and oil refineries. Studies have also been undertaken examining the GTFP of different industries using a meso perspective, such as investigations by Giang, Han, and Liu [8,9,10], who used the Super-SBM model (Slack Based Model) and the Malmquist index to measure the GTFP of manufacturing, agriculture, and logistics, as well as analyzed why the GTFP of these industries changed. By using a variety of mathematical models, the GTFP of micro-enterprises and meso-industries has been examined using an elaboration of research methods and the application of econometric models, thereby realizing a more realistic measurement of industrial economy and reversing the distortion of economic performance evaluation caused by ignoring resource consumption and environmental expense. However, these investigations have ignored analysis of spatial differentiation of industrial GTFP from a geographical perspective, which is not conducive to formulating strategies for sustainable and coordinated development of regional industries.

Current investigations on tourism productivity still use stagnant traditional total factor productivity (TFP) analysis, where measurement of tourism TFP only considers labor and capital indicators as input variables, and tourism revenue as expected output. Following the traditional measurement framework of TFP, the TFP of tourism hotels, travel agencies, tourism transportation, tourism attractions, and the tourism industry has been examined. Specifically, in the field of tourism hotel research, the TFP of hotels in the United States, Britain, and Morocco has been calculated based on the DEA model [11,12,13]. In terms of travel agency research, the majority of investigations have used the DEA model to analyze and explore factors affecting the TFP of travel agencies in Portugal, Turkey, and Spain [14,15,16]. In relation to travel traffic, Fernandes and Wanke [17,18] measured the TFP of airlines in Brazil and Nigeria, respectively, using the DEA model. Lee, Preda, and Ding, amongst others [19,20,21], examined the TFP of different types of tourist attractions (gymnasiums and forest parks, for example) using the DEA model and the Malmquist index, and proposed corresponding countermeasures to improve TFP. Finally, in terms of the tourist industry, Wang, Wu, and Zha [22,23,24] conducted empirical studies analyzing the TFP of tourism growth in China using either the DEA model or the SFA model (Stochastic Frontier Approach). All of these investigations are rooted in tourism TFP, and they measure the TFP of tourism segments (hotels, travel agencies, transportation, and attractions) or the tourist industry. However, current research on tourism TFP has not introduced energy consumption or carbon emissions that are closely related to the ecological environment into the tourism industry GTFP measurement framework. Current studies, therefore, are not able to accurately measure the level and quality of green tourism development, and it is even more difficult to truly realize harmonious development of tourism, resources, and the environment. Therefore, measurement and analysis of tourism GTFP in China have become the core issues.

Although significant progress has been made in the study of GTFP, limitations still exist. Firstly, the research object focuses on GTFP in manufacturing, agriculture, logistics, steel works, cement plants, and oil refineries. As studies on the GTFP of the tourist industry have not been undertaken, thus not being conducive to measuring the green development level of the tourism industry, it is therefore more difficult to measure the role of tourism in promoting ecological and environmental protection. Secondly, the research paradigm focuses on the elaboration and application of mathematical models; however, GTFP analysis of regional industries using a spatial perspective is lacking.

In view of this, we attempt to estimate carbon emissions and the consumption of energy related to China’s tourism industry, spanning 2006 to 2018, and measure the GTFP of the tourist industry over this time period using the DEA model and the Malmquist–Luenberger (ML) index. We will also examine regional characteristics of GTFP related to the tourism industry, clarifying the current situation and characteristics of tourism GTFP. These results will enable a space–time evolution law of tourism GTFP to be proposed, as well as provide targeted strategies for coordinating sustainable development of tourism in China.

## 3. Materials and Methods

### 3.1. Study Area

**Figure 1:**
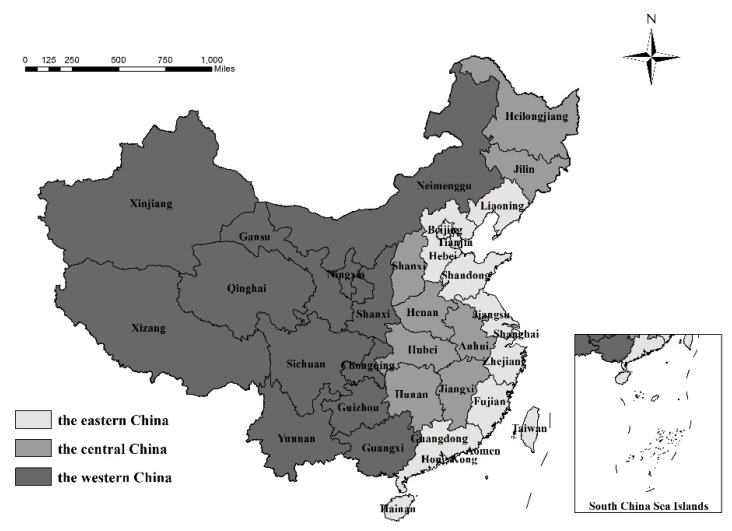
The three major regions in China. Note: The geographical base map is from the Ministry of Natural Resources of the P.R. China.

In this investigation, apart from Hong Kong, Macao, and Taiwan, 31 provinces were used (as shown in Figure 1). China can be subdivided into three regions (east, central, and west) using strategies of coordinated regional development and the geographical location of different provinces. The eastern region comprises 11 provinces, including Tianjin, Beijing, Liaoning, Hebei, Jiangsu, Shanghai, Zhejiang, Fujian, Shandong, Guangdong, and Hainan; 8 provinces make up the central region (Heilongjiang, Jilin, Shanxi, Anhui, Heilongjiang, Jiangxi, Henan, and Hubei); the western region comprises 12 provinces, including Chongqing, Sichuan, Guizhou, Tibet, Yunnan, Shaanxi, Ningxia, Gansu, Qinghai, Xinjiang, Guangxi, and Inner Mongolia.

### 3.2. Study Methods

#### 3.2.1. Calculation Method for Carbon Emissions and the Consumption of Energy Related to the Tourism Industry

Methods used to measure carbon emissions and the consumption of energy related to tourism have mainly included “top-down” and “bottom-up” methods. The top-down method, based on the perspective of production, relies on the national tourism satellite account to estimate carbon emissions and the consumption of energy related to tourism from regional macro statistics [25,26]. The bottom-up method initially uses data analysis of tourist destinations, estimates the relevant data of tourism behavior and enterprises, and calculates carbon emissions and the consumption of energy from the perspective of tourism [27,28]. As there is no energy consumption statistic item for tourism in the China Energy Statistical Yearbook, and China has not yet established a statistical monitoring system for carbon emissions, we therefore adopted the bottom-up method to estimate carbon emissions and energy consumption. Based on the research results of Howitt, Wei, and Shi [27,28,29] related to the bottom-up method, we initially divided tourism into three sectors (transport, accommodation, and activities) before estimating carbon emissions and the consumption of energy for each sector.

The estimation formula for carbon emissions and the consumption of energy related to tourism transportation was:

(1) $$C_{Tt}=\sum_{j=1}^{n}B_{j}\times K_{j}\times M_{jt}$$

where $C_{Tt}$ is carbon emissions and the consumption of energy of tourism transportation; *j* represents the four types of transportation (railway, highway, water transportation, and aviation); $K_{j}$ represents passenger turnover as the proportion of tourists in the *j*th mode of transport; and $M_{jt}$ is passenger turnover of the *j*th mode of transport in the t-year. After analyzing current research results [28,29], $K_{j}$ values for railway, water, highway, and aviation transport were 0.316, 0.138, 0.106, and 0.647, respectively, and energy consumption coefficients for these four modes of transport were taken as 1 *MJ*/passenger-km, 1.8 *MJ*/passenger-km, 2.4 *MJ*/passenger-km, and 2 *MJ*/passenger-km, respectively. According to the study of Shi et al. [29], carbon emission factors of the four transport types were 27 *g*/passenger-km, 133 *g*/passenger-km, 106 *g*/passenger-km, and 137 *g*/passenger-km, respectively.

The estimation formula for carbon emissions and the consumption of energy of tourism accommodation was:

(2) $$C_{Ht}=L_{t}\times Y_{t}\times D\times x$$

where $C_{Ht}$ is carbon emissions and the consumption of energy of tourism accommodation; $L_{t}$ is the room bed number in the t-year; $Y_{t}$ is average room occupancy rate in the t-year; *D* is set at 365 days; and x represents carbon emissions and the energy factor per bed per night. As per current investigations, x was set as 155 *MJ*/*p* visitor-night and 2.458 *g*/*p* visitor-night [28,29].

The estimation formula for carbon emissions and the consumption of energy related to tourism activities was:

(3) $$C_{At}=\sum_{r=1}^{n}W_{rt}\times B_{r}$$

where $C_{At}$ is carbon emissions and the consumption of energy for tourist activities; $W_{rt}$ is the number of tourists participating in *r*th tourism activities in the t-year; $B_{r}$ represents carbon emissions or the energy coefficient factor in the *r*th tourism activities; *r* is the collection of sightseeing, holidays, business, family visits, and other tourist activities. Based on current research results [28,29], energy consumption coefficients or carbon emission factors of tourism activities were 8.5 *MJ*/visitor and 417g/visitor, 12 *MJ*/visitor and 1670g/visitor, 26.5 *MJ*/visitor and 591g/visitor, 12 *MJ*/visitor and 786g/visitor, and 3.5 *MJ*/visitor and 172g/visitor for sightseeing, holidays, business, family visits, and other tourist activities, respectively.

In summary, the formula used to estimate carbon emissions and the consumption of energy related to tourism (using the bottom-up method) was:

(4) $$C_{t}=C_{Tt}+C_{Ht}+C_{At}$$

#### 3.2.2. Data Envelopment Analysis (DEA) Model and the ML Index

The data envelopment analysis (DEA) model was selected as the main tool to study tourism industry GTFP in China due to two advantages: firstly, it can avoid potential deviation caused by the preset expression form of the production function and the error term distribution hypothesis [30]; secondly, it is applicable to multi-input and multi-output systems [31]. The key to measuring industrial TFP based on the DEA model is the construction of a distance function. Chung et al. modified the Malmquist index to propose the Malmquist–Luenberger (ML) index, where a function using directional distance was formulated using the function of distance [32]. Compared with the Malmquist index, this index can measure positive outputs and reduce negative outputs, and it can better reflect green development demands [33]. Drawing on the research results of the GTFP from iron and steel, fossil energy, manufacturing, and other industries [6,7,8], we therefore adopted the DEA model based on the function of directional distance and the ML index to calculate tourism GTFP in China.

When resource and environment factors are included in the measurement framework of tourism GTFP, it is necessary to construct a production possibility set containing both expected and unexpected outputs. The directional distance function (DDF), indicating the possibility that expected outputs increase and unexpected outputs decrease in the production process, can be defined once the environmental technology set has been constructed. After the DDF has been solved, the ML index can be further constructed. According to the method proposed by Chung et al. [32], the dynamic concept of interphase is introduced, and the ML index from *t* period to *t* + 1 period is:

(5) $$ML_{t}^{t+1}=\frac{\left[1+D_{0}^{t+1}\left(x^{t},y^{t},m^{t},n^{t}\right)\right]}{[1+D_{0}^{t}\left(x^{t+1},y^{t+1},m^{t+1},n^{t+1}\right)}\times \;\sqrt{\left\{\frac{\left[1+{\vec{D}}_{0}^{t+1}\left(x^{t},y^{t},m^{t},n^{t}\right)\right]}{[1+D_{0}^{t}\left(x^{t},y^{t},m^{t},n^{t}\right)}\times \frac{\left[1+D_{0}^{t+1}\left(x^{t+1},y^{t+1},m^{t+1},n^{t+1}\right)\right]}{[1+D_{0}^{t}\left(x^{t+1},y^{t+1},m^{t+1},n^{t+1}\right)}\right\}}$$

In this formula, the indices of TE (technological progress) and EFF (technical efficiency) were decomposed from the ML index. For the EFF index, it can also be further deconstructed into the indices of PE (pure technical efficiency) and SE (scale efficiency). Therefore, the ML index can be expressed as:

(6) $$ML_{t}^{t+1}=TE\times EFF=TE\times PE\times SE$$

where TE indicates the production front speed from the *t* phase to the *t* + 1 phase, reflecting the amount of production technology innovation; EFF measures the extent to which the production system catches up with the boundary of production possibility from *t* to *t* + 1 period, and represents the degree of effective utilization of resources; PE reflects system efficiency and management level efficiency (i.e., the improvement of the management system results in the improvement of the overall efficiency level); and SE shows the degree to which scale expansion can improve efficiency at a given level of regulation and management. The four indices are all bounded by 1, with a value >1 indicating technological progress or efficiency improvement, and vice versa.

### 3.3. Index Selection and Data Sources

Given the three characteristics of data selection of the DEA model (collectability, relevance, and reliability), and drawing on the research results of the GTFP index system from manufacturing, agriculture, logistics, and other industries [8,9,10], we constructed a GTFP system for the tourism industry based on the input–output principle. The input system includes capital, labor, and energy input, represented by the net value of fixed assets, the number of employees, and consumption of energy in the tourism industry. The output system includes expected output and nonexpected output, represented by revenue and carbon emissions related to tourism. By taking data availability and continuity into account, we chose the intertemporal panel data of investment and output for the tourist industry in China from 2006 to 2018, thus measuring tourism GTFP. Input and positive output indicators of tourism included the net value of fixed assets, employee numbers in the tourism industry, tourism revenue, and other indicators from the China Tourism Statistical Yearbook. Data involved in carbon emissions and the estimation of consumption of energy for the tourism industry were derived from the China Traffic Statistical Yearbook (tourist turnover of railways, highways, water transportation, and aviation), China Tourism Statistical Yearbook (occupancy rates and bed numbers), and the Tourism Sampling Survey (relevant data of tourist activities).

## 4. Research Process and Results

### 4.1. Temporal Features and Spatial Differences of Carbon Emissions and the Consumption of Energy

#### 4.1.1. Temporal Features of Carbon Emissions and the Consumption of Energy

**Figure 2:**
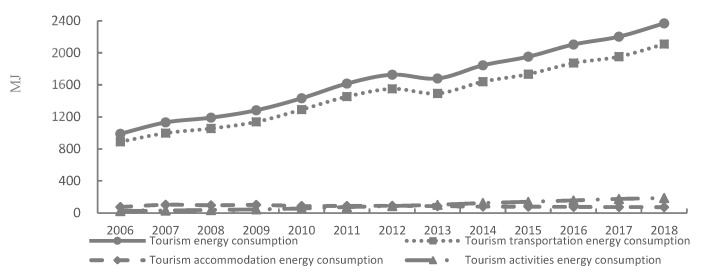
The estimated consumption of energy in tourism industry in China, 2006 to 2018. Note: Data is calculated and processed from this article.

**Figure 3:**
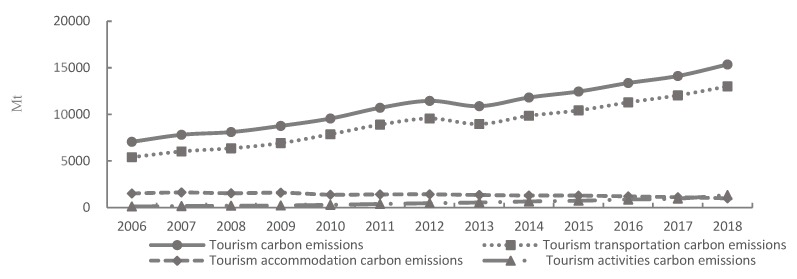
The estimated carbon emissions in tourism industry in China, 2006 to 2018. Note: Data is calculated and processed from this article.

Consumption of energy shown in Figure 2 and carbon emissions shown in Figure 3 of China’s tourism industry recorded increasing trends from 2006 to 2018. Specifically, carbon emissions and the consumption of energy increased from 7056.25 million tons and 989.25 MJ to 15,351.70 million tons and 2368.35 MJ from 2006 to 2018 (shown in Figure 2 and Figure 3), recording a growth rate of 10% and 12% annually, respectively. From the perspective of temporal change, the fastest rate of increase was recorded in 2011. Rapid growth in carbon emissions and the consumption of energy can be related to two main reasons. Firstly, with the onset of mass tourism and an increasing demand by Chinese tourists to travel, the scale of the tourism industry in China rapidly expanded. For example, domestic and foreign tourists reached 5.539 billion and 141.2 million in 2018 [34], respectively. Secondly, the tourism industry in China is still in the development and transformation stage; the mode of development is relatively extensive, having a large dependency on capital, resources, labor, and other inputs, which are not conducive to promoting the sustainable development capacity for the tourism industry in China [35].

Results indicate that carbon emissions and the consumption of energy related to transport listed in Table 1 are increasing, and that the proportion of energy consumed and carbon emissions remain around 89%. Due to continuous expansion of tourism in China and the improvement of the transportation infrastructure, the proportion of carbon emissions and energy consumed related to the transport of tourists will continue to be significant. Carbon emissions and the consumption of energy associated with accommodation (as shown in Table 1) have recorded downward trends, with accommodation going from being the second largest contributor to the third largest. This change indicates that the accommodation industry has made remarkable achievements in energy conservation and reducing emissions. The most important reason accounting for the downward trends is that China has intensified efforts to promote the construction of green hotels through institutional formulation and policy improvement. Carbon emissions and the consumption of energy associated with tourist activities have rapidly increased (as shown in Table 1); for example, carbon emissions and energy consumption related to tourism activities in 2018 were 10 times and 7 times higher than those in 2006, respectively. With the implementation of paid vacation time, the increase in the proportion of middle-income groups, and the promotion of tourism supply-side reform, tourism activities have become an essential way of life for people. However, as the development mechanism of green tourism is not perfect, the promotion of tourism technology with a reduced carbon footprint is weak, and the concept of civilized tourism has not been widely popularized, tourist activities have therefore resulted in higher levels of carbon emissions and the consumption of energy.

#### 4.1.2. Space Characteristic of Carbon Emissions and the Consumption of Energy

**Figure 4:**
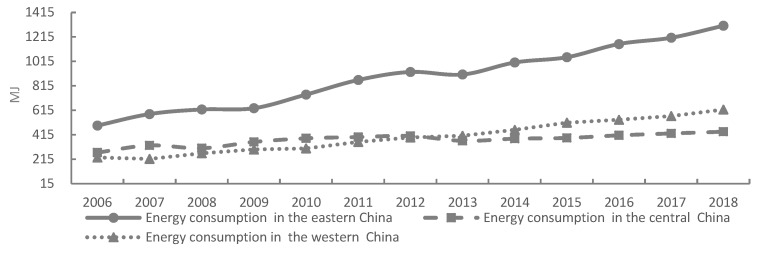
Estimation of the energy consumption of tourism by regions of China, 2006 to 2018. Note: Data is calculated and processed from this article.

**Figure 5:**
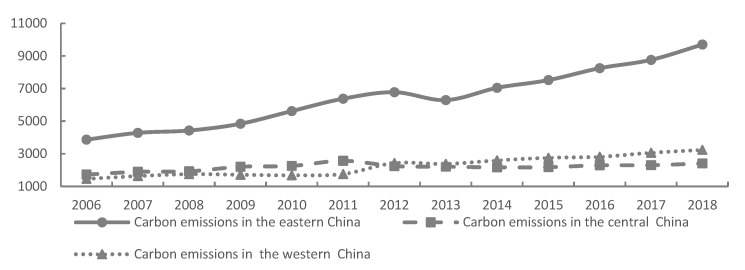
Estimation of the carbon emissions of tourism by regions in China, 2006 to 2018. Note: Data is calculated and processed from this article.

Results for carbon emissions and the consumption of energy associated with tourism in the three regions (east, central, and west) of China indicated notable differences (see Figure 4 and Figure 5). In particular, economic growth associated with tourism, the high density and quality of tourist attractions, and the optimal accessibility of tourism traffic resulted in large-scale tourism in the eastern region, as well as an accumulation of environmental problems. The growth trend for both factors was greatest in the eastern region, with the carbon emissions and energy consumption of tourism in the eastern region reaching 9705.36 million tons and 1406.58 MJ in 2018, respectively. Energy consumption and carbon emissions in the central region presented a state of "growth–reduction–growth" (see Figure 4 and Figure 5). From 2006 to 2011, driven by the Strategy of Rise of Central China, the scale of tourism in this region rapidly expanded, which further accelerated growth in both factors. From 2012 to 2013, these factors reduced, indicating that the excessive input of factors resulted in the phenomenon of "diseconomy of scale". After 2014, this region comprehensively promoted the construction of “Beautiful China” and ecological civilization, with an increase in focus on economic, societal, and environmental development, resulting in a decline in the growth rate of carbon emissions and the consumption of energy. In western China, growth rates of these factors were increasing at an accelerated rate. Policies such as the Western Development Strategy and the "One Belt and One Road" strategy resulted in the government, society, and enterprises attaching great importance to the tourism industry in this region. However, the mode of development in the tourist industry in the western region is still relatively extensive, and the implementation of policies to reduce emissions and conserve energy are relatively weak, resulting in the continuous acceleration of carbon emissions and the consumption of energy. Here, carbon emissions and energy consumption in western China recorded an annual growth rate of 9% and 12%, respectively.

### 4.2. Temporal Evolution and Regional Differences of GTFP of China’s Tourism Industry

#### 4.2.1. Temporal Evolution of GTFP of China’s Tourism Industry

**Figure 6:**
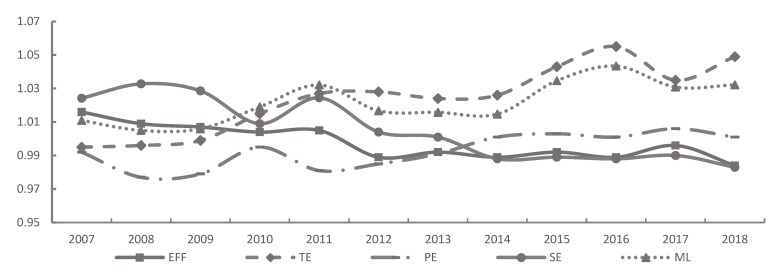
The indices of ML, EFF, PE, SE, and for the three regions in China, 2006 to 2018. Note: Data is calculated and processed from this article.

The ML index of tourism GTFP and its decomposition terms from 2007 to 2018 were calculated using the estimated values of tourist carbon emissions and the consumption of energy. The mean ML index peaked at 1.022 (as shown in Figure 6), indicating a relatively high level of green development performance. This was closely related to the development of green tourism products, the application of new technologies in tourism, and the green transformation of tourism in the context of high-quality development. However, it is worth noting that although tourism GTFP in China generally increased, the rate of increase was not large, which to some extent indicates that the extensive development mode causes the green development of tourism in China to be slow. When the ML index was separated into the TE and EFF indices, it was identified that the EFF index was >1 from 2007 to 2011, and the TE index was >1 from 2008 to 2012 (as shown in Figure 6). This result indicates that development of green tourism in China had been gradually transformed from being driven by technological efficiency to being driven by technological progress. The decline of the EFF index (as shown in Figure 6) indicates that the catch-up effect was weakened, possibly resulting in the gap of tourism GTFP among the 31 provinces to increase. By dividing the EFF index into the PE index and SE index, we found that the SE index was >1 between 2006 and 2015 (as shown in Figure 6), indicating that technical efficiency improvement during this period mainly depended on the expansion of scale rather than an improvement in tourism management level or the tourism management system. After 2014, the PE index gradually exceeded 1 (see Figure 6), indicating that the tourism industry gradually focused on upgrading the industry by improving the allocation efficiency of resources and improving the tourism management system.

#### 4.2.2. Spatial Pattern of GTFP for the Tourism Industry in China

**Figure 7:**
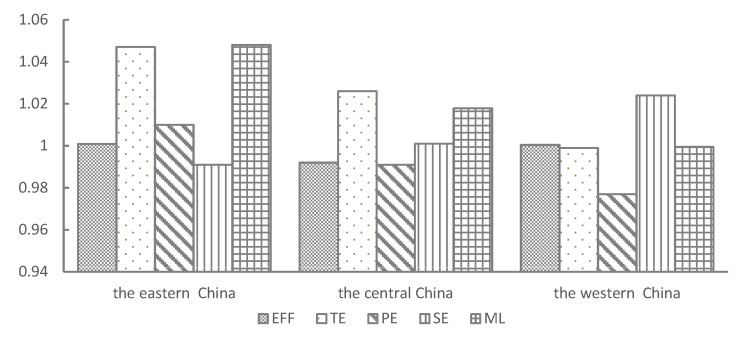
The indices of ML, EFF, PE, SE, and for the three regions in China, 2006 to 2018. Note: Data is calculated and processed from this article.

During the study period, a large spatial difference in GTFP for the tourism industry in China was recorded (as shown in Figure 7). In the three regions, the mean ML index value in the eastern region was 1.046, this being the highest of the regions. In addition, average TE and EFF index values were both >1 (as shown in Figure 7), indicating that tourism green development in this region depended on technological progress and technological efficiency. Furthermore, mean PE index was >1 in the eastern region, indicating that this region attached great importance to the reform and innovation of tourism management systems and mechanisms, such as taking the lead in promoting the establishment of national parks, exploring ways to establish a green tourism consumption system, and trying to build the appraisal platform of green development performance of tourism enterprise. Although mean ML and TE index values for the tourism industry in the central region were both >1, the PE index was <1 (as shown in Figure 7). This result indicated that tourism green development performance in this region was relatively good, however, the innovation degree of the tourism management system was insufficient and the factor input ratio was poor, resulting in a redundancy of tourism resource input factors and the decline in marginal productivity of tourism resources. Results for the western region recorded mean ML, TE, and PE index values <1, however, mean values for EFF and SE indices were >1 (see Figure 7), indicating that this region had become an area with depressed green development. Green development of the tourism industry mainly depended on improving scale efficiency, a mode of scale expansion that is unsustainable.

**Figure 8:**
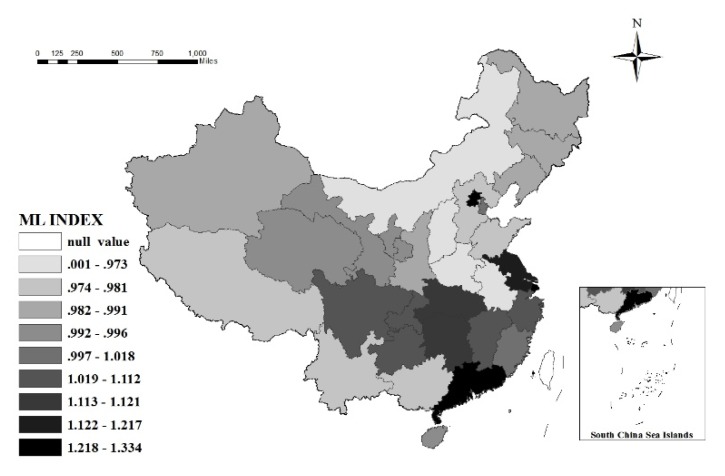
The spatial distribution of the ML index in 31 provinces in China during 2006 to 2018. Note: The geographical base map is from the Ministry of Natural Resources of the P.R. China.

In addition, average ML index values for Beijing, Shanghai, Jiangsu, Guangdong, and other provinces in the eastern region (as shown in Figure 8) were the highest in China. These provinces mainly rely on technological innovation, introduction and imitation to promote green tourism, and sustainable development. These provinces have therefore become the core growth poles of green tourism development in China. With the help of regional integration policies, improvement of the high-speed rail network, and the establishment of a talent flow mechanism, these core growths will continue to strengthen the spatial connection of tourism GTFP with other provinces in China, and further promote the green development level in China. Average ML index values in Hubei, Hunan, Jiangxi, Chongqing, Sichuan, and other provinces in the central or western region were all >1 (as shown in Figure 8), being secondary growth poles for green tourism development in China, therefore leading, demonstrating, and driving the central and western regions.

## 5. Discussion and Conclusions

In this investigation, we used a bottom-up method to estimate carbon emissions and the consumption of energy in China’s tourist industry from 2006 to 2018. A tourist GTFP framework was established using the DEA model and the ML index. At the same time, GTFP temporal and spatial patterns were also analyzed. In summary, the main research conclusions of this study were as follows.

Firstly, tourism industry carbon emissions and energy consumption in China increased year by year, and there is still room for further increases. This finding is consistent with those of, amongst others, Han, Zheng, and Wang, who recorded that rapid expansion of tourism in China resulted in an increase in carbon emissions and energy consumption, and environmental issues related to these factors will become increasingly significant [1,36,37]. In addition, it has been recorded that tourism industry carbon emissions and energy consumption in both developed countries (France, Switzerland, and Australia) and developing countries (Pakistan and Malaysia) are still increasing with the continuous expansion of tourism scale [38,39,40,41,42]. In the tourism industry segments, carbon emissions and energy consumption related to the transportation of tourists account for more than 80%, a finding that is in agreement with the results of [26,38,43,44,45]. Results also indicated that carbon emissions and energy consumption associated with tourism activities and accommodation change with each other. From the perspective of regional differentiation, spatial distributions of carbon emissions and energy consumption are unbalanced, having characteristics of generally being high in the east and low in the west.

Secondly, tourism GTFP represented a good development trend with small fluctuations, highlighting that green tourism development in China has achieved a good level of performance. Technological advances have also gradually become the core endogenous driving force promoting the development of green tourism. Technological efficiency, however, has continuously decreased due to technical efficiency being squeezed by technological progress. Moreover, improvement in technical efficiency in China’s tourism industry has shifted from relying on scale expansion to depending on structural adjustment and industrial upgrading system reform, indicating that the extensive development mode has been gradually abandoned by the tourist industry. Therefore, it is evident that the internal law of tourism GTFP development in China is in accordance with the economic trajectory in China. That is, technological innovation is gradually driving the replacement of large-scale, high-input extensive development by high-quality economic and green development [46].

Thirdly, from the perspective of spatial differentiation, there are significant spatial differences in tourism GTFP. The overall spatial pattern of strong in the east and weak in the west has not significantly changed. Specifically, the development of green tourism in the eastern region is driven by technological progress and technological efficiency, and the central region mainly depends on technical progress, with weak technical efficiency. However, green tourism development performance in the western region is not ideal, and its driving force mainly relies on technical efficiency, with scale expansion still the main mode of tourism development. In addition, based on the provincial perspective, a few provinces in the eastern region, such as Beijing, Shanghai, Jiangsu, and Guangdong, have the highest level of tourism green development in China; Hubei, Hunan, Jiangxi, Chongqing, Sichuan, and other provinces in central or western regions have a high level of green tourism development, which can be the core or secondary growth poles of green tourism development in China.

Finally, the main way to improve the GTFP of tourism in China is to promote low-carbon, coordinated, and sustainable tourism development. Recommendations include: (i) China should continue to strengthen innovation and the introduction of tourism energy-saving and environmental protection technologies, and extend the promotion of technologies with a low-carbon footprint tourism segment. These developments will curb excessive growth of energy consumption and increased carbon emissions related to tourism. (ii) It is vital to adhere to the concept of green development resulting in "clear waters and green mountains mean golden mountains", and the Chinese government should promote reformation of the tourism development system and mechanism. (iii) Targeted strategies for developing green tourism in the eastern, central, and western regions should focus on continued technological innovation, actively introducing, absorbing, and utilizing low-carbon development technology, and paying more attention to the reform of the tourism management system. (iv) The spatial spillover effect of core growth poles or secondary growth poles should be fully recognized, thereby radiating and promoting green tourism development in other provinces in China.

In this study, we not only attempted to construct a framework for measuring tourism GTFP, which includes carbon emissions and energy consumption, we also analyzed the internal law of GTFP change for tourism in China using spatial and temporal factors. This study, therefore, is innovative to some extent in its approach. However, there are still four shortcomings in this study which require further analysis. Firstly, only carbon emissions were selected to represent the nonexpected output in this research, which is one-sided to some degree. In subsequent studies, it is necessary to construct indicators of nonexpected output from multiple dimensions such as wastewater, waste gas, and solid waste. Secondly, the bottom-up method mainly takes into account tourism transportation, accommodation, and tourism activities to estimate carbon emissions and energy consumption of tourism. However, as a comprehensive industry, tourism is composed of different sectors like food, shelter, transportation, travel, purchase, and entertainment, all of which directly or indirectly contribute to energy consumption and carbon emissions. Therefore, it is necessary to improve the formula of energy consumption and carbon emissions of the tourism industry in future studies. Thirdly, although we used environmental pollution as a nonexpected output, there is still some controversy regarding whether environmental pollution should be included in the input index or the nonexpected output in investigations. Subsequent studies, therefore, can combine different research scenarios and measurement methods to further examine which option is better. Finally, due to space limitations, factors influencing tourism GTFP were not analyzed. Therefore, we could not construct the influence mechanism, and our investigation constructed a dynamic spatial econometric analysis of impact mechanisms related to tourism GTFP in China from the perspective of spatial heterogeneity.

## Figures and Tables

**Table 1:** The estimations of the carbon emissions and the consumption of energy in tourism industry in China, 2006 to 2018.

	Tourism Transportation (Mt/MJ)		Tourism Accommodation (Mt/MJ)		Tourism Activities (Mt/MJ)	
	Carbon Emissions	Energy Consumption	Carbon Emissions	Energy Consumption	Carbon Emissions	Energy Consumption
2006	5406.25	888.81	1520.29	75.21	129.71	25.23
2007	6024.13	997.60	1626.61	102.57	161.57	30.83
2008	6361.16	1055.53	1541.50	97.21	199.77	37.71
2009	6923.07	1137.50	1606.86	101.33	234.29	44.16
2010	7873.76	1290.39	1387.55	87.50	295.35	55.19
2011	8898.05	1452.95	1415.40	89.25	393.51	73.33
2012	9558.88	1550.01	1427.93	90.04	468.97	86.80
2013	8972.85	1492.79	1357.35	85.59	551.46	101.60
2014	9847.96	1639.41	1301.23	80.16	665.06	124.09
2015	10424.99	1733.29	1292.06	78.95	740.21	139.29
2016	11287.10	1872.19	1206.63	76.47	869.74	155.95
2017	12037.19	1952.37	1120.71	74.35	967.95	175.36
2018	13001.65	2108.52	1001.29	73.25	1348.76	186.58

Note: Data is calculated and processed from this article.
